# *PLK*:Δ*gra9* Live Attenuated Strain Induces Protective Immunity Against Acute and Chronic Toxoplasmosis

**DOI:** 10.3389/fmicb.2021.619335

**Published:** 2021-03-11

**Authors:** Jixu Li, Eloiza May Galon, Huanping Guo, Mingming Liu, Yongchang Li, Shengwei Ji, Iqra Zafar, Yang Gao, Weiqing Zheng, Paul Franck Adjou Moumouni, Mohamed Abdo Rizk, Maria Agnes Tumwebaze, Byamukama Benedicto, Aaron Edmond Ringo, Tatsunori Masatani, Xuenan Xuan

**Affiliations:** ^1^State Key Laboratory of Plateau Ecology and Agriculture, Qinghai University, Xining, China; ^2^College of Agriculture and Animal Husbandry, Qinghai University, Xining, China; ^3^National Research Center for Protozoan Diseases, Obihiro University of Agriculture and Veterinary Medicine, Obihiro, Japan; ^4^The Collaboration Unit for Field Epidemiology of State Key Laboratory for Infectious Disease Prevention and Control, Jiangxi Provincial key Laboratory of Animal-origin and Vector-borne Diseases, Nanchang Center for Disease Control and Prevention, Nanchang, China; ^5^Department of Internal Medicine and Infectious Diseases, Faculty of Veterinary Medicine, Mansoura University, Mansoura, Egypt; ^6^Transboundary Animal Diseases Research Center, Joint Faculty of Veterinary Medicine, Kagoshima University, Kagoshima, Japan

**Keywords:** *Toxoplasma gondii*, toxoplasmosis, live attenuated vaccine, *PLK*:Δ*gra9*, protective immunity

## Abstract

Toxoplasmosis is a zoonotic parasitic disease caused by the obligate intracellular protozoa *Toxoplasma gondii*, which threatens a range of warm-blooded mammals including humans. To date, it remains a challenge to find safe and effective drug treatment or vaccine against toxoplasmosis. In this study, our results found that the development of a mutant strain based on gene disruption of dense granule protein 9 (gra9) in type II PLK strain decreased parasite replication *in vivo*, severely attenuated virulence in mice, and significantly reduced the formation of cysts in animals. Hence, we developed an immunization scheme to evaluate the protective immunity of the attenuated strain of Δ*gra9* in type II PLK parasite as a live attenuated vaccine against toxoplasmosis in the mouse model. Δ*gra9* vaccination-induced full immune responses characterized by significantly high levels of pro-inflammatory cytokine interferon gamma (IFN-γ) and interleukin-12 (IL-12), maintained the high *T. gondii*-specific immunoglobulin G (IgG) level, and mixed high IgG1/IgG2a levels. Their levels provided the complete protective immunity which is a combination of cellular and humoral immunity in mouse models against further infections of lethal doses of type I RH, type II PLK wild-type tachyzoites, or type II PLK cysts. Results showed that Δ*gra9* vaccination proved its immunogenicity and potency conferring 100% protection against acute and chronic *T. gondii* challenges. Together, Δ*gra9* vaccination provided safe and efficient immune protection against challenging parasites, suggesting that *PLK*:Δ*gra9* is a potentially promising live attenuated vaccine candidate.

## Introduction

Toxoplasmosis is a zoonotic parasitic disease caused by the obligate intracellular protozoan *Toxoplasma gondii* (Montoya and Liesenfeld, 2004; Saadatnia and Golkar, 2012). *T. gondii* has the ability to infect all nucleated cells, and thus, has a broad host range of warm-blooded mammals, including humans (Loh et al., 2019). One-third of the global population is estimated to have *T. gondii* infection, most of which are asymptomatic in immunocompetent people, but causes severe complications in immunocompromised individuals and pregnant women (Tenter et al., 2000; Innes, 2010; Robert-Gangneux and Darde, 2012; Le Roux et al., 2020). Additionally, reproductive problems, i.e., abortion and stillbirth, induced by *T. gondii* infection in livestock presents a grave challenge to the animal industry (Robert-Gangneux and Darde, 2012; Wang et al., 2019). The ensuing public health problems and agricultural economic losses necessitate the search for and development of safe and effective drug treatments and vaccines against toxoplasmosis in humans and animals.

Despite unsparing research efforts in recent decades, treatment and vaccine options against toxoplasmosis are still limited, owing to the unique characteristics of *T. gondii*. For instance, *Toxoplasma* infection has multiple routes of transmission in humans or animals. One route is ingestion of raw or undercooked meat containing tissue cysts with bradyzoites which transmits the parasite to humans. Although a combination of pyrimethamine and sulfadiazine or other compounds has been used to treat active toxoplasmosis in animals or humans (Alday and Doggett, 2017; Dunay et al., 2018), there is no significant therapeutic efficiency on the bradyzoite residing within tissue cysts. Furthermore, *T. gondii* develops complex population structures, in which North America and Europe strains are classified into three major clonal lineages, type I, II, and III (Howe and Sibley, 1995; Loh et al., 2019). The composition of these complex strains will inevitably bring new challenges to the control of toxoplasmosis because of the different proliferative ability and degree of pathogenicity in mouse models. Therefore, due to the current unsatisfactory status in drug treatment of toxoplasmosis, such as the inability of eliminating tissue cysts, the development of a vaccine to control *Toxoplasma* infections caused by different strains and contracted through multiple routes has been a priority.

Several studies on *T. gondii* vaccines have been done and reported in animal models. Although compared with nucleic acid vaccines (Li et al., 2015; Zhang et al., 2015; Lu et al., 2017) and recombinant protein vaccines (El Bissati et al., 2014; Tanaka et al., 2014; Wang et al., 2014a,b), the higher protection against acute or chronic *T. gondii* infection provided by live attenuated vaccines (Fox and Bzik, 2015; Lagal et al., 2015; Abdelbaset et al., 2017; Wang et al., 2017, 2018; Xia et al., 2018b; Yang et al., 2019; Liang et al., 2020) was highlighted both in short- and long-term vaccination using different strains in the mouse model. However, the safeness of these mutants still needs to be tested in animal models. With the advent of the genomic era, the widespread application of the CRISPR/Cas9 system has permitted precise and efficient genetic manipulations in *T. gondii*, such as gene editing and gene deletion resulting in attenuated strains which can be functionally selected (Shen et al., 2017). Advantages of this system facilitate the development of a live attenuated vaccine with reduced virulence but retaining its ability for limited replication in order to induce an immune response, which is considered as the ideal vaccine for resisting toxoplasmosis (Wang et al., 2019).

Dense granule proteins (GRAs) play major functions within the structural formation of the parasitophorous vacuole (PV) and the cyst wall of *T. gondii* (Guevara et al., 2019). One of the GRAs, the dense granule protein 9 (GRA9), has been characterized in *T. gondii*. Recent studies reported that GRA9 was investigated as one of the intravacuolar-network-associated GRAs during cyst development *in vitro*, and loss of gra9 in type II Prugniaud (Pru) strain induced severe defects in the development of chronic-stage cysts *in vivo* (Fox et al., 2019; Guevara et al., 2019). More so, our previous study revealed that disruption of gra9 gene in *T. gondii* type II PLK strain significantly reduced the growth of tachyzoites *in vitro* (Guo et al., 2019). In the current study, we confirmed that the development of a mutant strain based on gene disruption of gra9 in type II PLK strain decreased parasite replication *in vivo*, severely attenuated virulence in mice, and significantly reduced the formation of cysts in animals. These suggest that Δ*gra9* could be considered a vaccine candidate. Hence, we developed an immunization scheme to evaluate the protective immunity of the attenuated strain of Δ*gra9* in type II PLK parasite as a live attenuated vaccine against toxoplasmosis in the mouse model. Results showed that Δ*gra9* vaccination proved its immunogenicity and potency with 100% protection against acute and chronic *T. gondii* challenge infections.

## Materials and Methods

### Animals and Parasite Strains

The recommendations in the Guide for the Care and Use of Laboratory Animals of Obihiro University of Agriculture and Veterinary Medicine, Japan were strictly followed. The protocol of this study was approved by the Committee on the Ethics of Animal Experiments at the Obihiro University of Agriculture and Veterinary Medicine, Japan (permission numbers: 190246). Six-week-old female BALB/c mice were purchased from Clea Japan. All animals were housed in the animal facility of the National Research Center for Protozoan Diseases, Obihiro University of Agriculture and Veterinary Medicine, with adequate temperature (25 ± 2°C) and luminosity (12-h light and 12-h dark) under specific pathogen-free conditions, and free access to food and water. All animals were used at least one week after habituation.

All procedures of pathogen experiments were carried out according to the guidelines of Obihiro University of Agriculture and Veterinary Medicine (permission number: 2018728). In this study, *T. gondii* type I (RH strain with hypoxanthine-xanthine-guanine phosphoribosyl transferase deficiency), and type II (PLK, which is a clone of ME49 strain) strain (Kirkman et al., 2001) were used. Mutant *PLK*:Δ*gra9* attenuated strain with green fluorescent protein (GFP) was generated and cultured in our laboratory, which has been passed for more than 250 generations (Guo et al., 2019). All parasites were cultured in human foreskin fibroblast (HFF) cells maintained in Dulbecco’s modified Eagle’s medium (DMEM, Sigma), as previously described (Li et al., 2020).

### Bradyzoite Differentiation *in vitro* of Δ*gra9* Strain

*Toxoplasma gondii* PLK wild-type (WT) or *PLK*:Δ*gra9* parasites were cultured in RPMI 1640 medium supplemented with 50 mM HEPES and 1% fetal bovine serum, pH 8.2, ambient CO_2_ for 4 days, as previously described (Xia et al., 2018a). The parasites (2 × 10^5^ per well) forced to egress were allowed to invade cell monolayer (1 × 10^5^ per well) for 3 h, then washed, and each culture was allowed to grow under bradyzoite-inducing conditions for 24, 48, 72, and 96 h, as described above. Subsequently, samples were fixed by 4% paraformaldehyde. After permeabilizing with 0.3% Triton X-100/phosphate-buffered saline (PBS), samples were incubated with rabbit anti-SAG1 polyclonal antibody diluted to 1:500. The Alexa Fluor 594-conjugated goat anti-rabbit IgG (Life Technologies, Inc., United States) and *Dolichos biflorus Agglutinin*, FITC Conjugate (DBA-FITC) (Vector Laboratories, United States) were used to detect primary antibody and cyst wall. Samples were examined using the All-in-one Fluorescence Microscope (BZ-900, Keyence, Japan). Conversion rates were counted in at least 100 vacuoles, wherein DBA-FITC (green) was used for cyst wall staining and anti-SAG1 (red) antibody was used for tachyzoite marker. All assays were conducted in triplicate and repeated at least three times.

### Mutant Δ*gra9* Parasite Infection Tests in Mice

To determine the virulence of *PLK*:Δ*gra9* in animals, six BALB/c mice were injected with a lethal dose (freshly egressed tachyzoites, 1 × 10^5^ per mouse) by intraperitoneal injection (i.p.). Daily observations of body weight and clinical signs were noted. Clinical scores ranged from 0 to 10, denoting presence of no signs or all signs, respectively. Evaluated clinical signs included hunching, piloerection, worm-seeking, behavior, ptosis, sunken eyes, ataxia, the latency of movement, deficient evacuation and touch reflexes, and lying on belly (Leesombun et al., 2016). Surviving mice were monitored for 30 days and blood was drawn at day 30 to confirm infection by an ELISA. Tissues were collected to determine parasite burdens through an examination of *TgB1* gene by quantitative PCR (qPCR). *T. gondii* PLK strain was used as control.

To test the cyst formation in animals, 10^3^ tachyzoites of *PLK*:Δ*gra9* was used to infect four 7-week-old female BALB/c mice by i.p. Mice were monitored for 35 days, and sera from the blood samples were tested to confirm infection by ELISA. Fresh brain cysts were isolated from each positive mouse brain homogenates, and the number of cysts was estimated by DBA-FITC staining, as previously described (Huskinson-Mark et al., 1991). *T. gondii* PLK WT strain was used as control.

To test the difference in immune response between PLK and *PLK*:Δ*gra9*-infected mice, the different doses of parasites including 10^3^, 10^4^, or 10^5^ were used to infect mice and clinical signs or *T. gondii*-specific IgG levels were noted. Moreover, sera of 10^3^ *T. gondii* PLK or *PLK*:Δ*gra9*-injected mice as above, were collected at day 7 or 30 post-infection to determine *T. gondii*-specific IgG levels using 0.5 μg/ml soluble PLK tachyzoite antigens coated by ELISA assay, and cytokine productions such as interleukin 12 (IL-12), interleukin 10 (IL-10), and interferon-gamma (IFN-γ) were tested using ELISA kits (Thermo Fisher Scientific, United States) according to the manufacturer’s recommendations. Furthermore, the difference in cytokine productions by splenocytes after *T. gondii* antigen stimulation was determined in infected mice with PLK or *PLK*:Δ*gra9* parasite at day 35 post-infection, as follows.

### Vaccination of Mice and Immunogenicity Measurements

Mice were either vaccinated once with 10^3^ freshly harvested *PLK*:Δ*gra9* tachyzoites or mock-vaccinated in a total of 200 μl PBS i.p. 30 and 70 days post-infection (dpi), then, sera were collected to test total *T. gondii*-specific IgG and IgG subclasses (IgG1 and IgG2a) levels. Briefly, the 96-well ELISA plates were coated with 0.5 μg/ml soluble tachyzoite antigens of PLK parasites diluted in coating buffer (0.05 M Carbonate-Bicarbonate, pH 9.6) and incubated at 4°C overnight. The ELISA plates were washed by PBS-T (0.05% Tween-20) three times, and then blocked with 3% BSA, then washed once. Collected sera were diluted by 1:50 and incubated for 1 h at 37°C. The plates were washed with PBS-T six times, the HRP conjugated goat anti-mouse IgG, IgG1 and IgG2a secondary antibodies were added and incubated for another 1 h at 37°C. After washing six times, ABTS (2, 2′-Azinobis [3-ethylbenzothiazoline-6-sulfonic acid]-diammonium salt) substrate was used to develop the reaction and measure the results at OD 415 nm. All samples were analyzed three times. Meanwhile, cytokine productions IL-12, IL-10, and IFN-γ were determined using ELISA kits according to the manufacturer’s recommendations, as above.

### Cytokine Productions of Splenocytes by *T. gondii* Antigens Stimulation After Vaccination

Vaccinated mouse spleens were isolated to test stimulating cytokine productions of splenocytes at 70 days post-vaccination (dpv), and unvaccinated mouse spleens were also collected to use as controls. In brief, the splenocytes were washed three times with RPMI 1640 (Sigma, United States), and hemolyzed in a lysing buffer (0.83% NH_4_Cl and 0.01 M Tris-HCl, pH 7.2) for 5 min, then washed with RPMI 1640. The viability of the splenocytes was determined by trypan blue staining. A total of 3 × 10^5^ viable splenocytes each well of 96-well cell culture plates were plated and cultured in RPMI 1640 supplemented with 20% FBS maintained 24 h. The final concentration of 50 μg/ml of *T. gondii* soluble antigens (TSA) of PLK parasites were used to stimulate cytokine productions for 3 days. Then supernatants from each well were harvested for cytokine level measurements, as above. For negative and positive controls, the same number of splenocytes was also plated into 96-well cell culture plates at the same time and stimulated with RPMI 1640 with 20% FBS only or 5 μg/ml concanavalin A (Sigma, United States) for 3 days, respectively. Each spleen-harvested splenocytes were plated in at least three wells for TSA, negative and positive control, and each supernatant sample was tested three times.

### Protective Immunity Against *T. gondii* Challenges

BALB/c mice were vaccinated with 10^3^ tachyzoites of *PLK*:Δ*gra9* through i.p. At 70 dpv, mice were challenged with 10^3^ type I RH or 10^5^ type II PLK tachyzoites by i.p., or 50 PLK cysts by oral administration (six mice for each group). Unvaccinated mice with same ages and numbers were used as control and infected with the same doses and routes. All challenged mice were monitored for another 30 days for tachyzoite-challenge or 35 days for cyst-challenge infections to record daily body weights, clinical signs, and survival rates in detail. Meanwhile, at 7 days tachyzoite post-challenges or 14 days cyst post-challenges, peritoneal fluid and serum samples of all experimental mice were collected to test cytokine productions. Parasite burdens were examined in peritoneal fluids during acute challenges at day 7 post-challenges. Furthermore, for chronic infection, the number of cysts in survival mouse brains was detected as above at 35 days cyst post-challenges.

### Passive Immunization Test of Δ*gra9*-Vaccinated Sera

BALB/c mice were infected with 10^5^ type II PLK tachyzoites through i.p. At the day 0 and 3 post-infection, the 100 μl sera from naïve mice or the day 70 after *PLK*:Δ*gra9-*vaccinated per mouse were administered into WT parasite infected mice by intraperitonea injection, which includes four mice for naïve sera as negative control, and five mice for *PLK*:Δ*gra9-*vaccinated sera as test group. Survival rates were recorded. Parasite burdens were examined in peritoneal fluids at day 5 or 7 post-infection by qPCR as follows to evaluate parasite growth under passive immunization.

### DNA Isolation and Quantitative PCR (qPCR) Detection of Parasite Burdens in Infected Mice

DNA was extracted from the tissues or peritoneal fluid of parasite-challenged mice by DNeasy Blood & Tissue Kit (Qiagen, Germany), according to the manufacturer’s instructions. The 50 ng DNA was then amplified with primers specific to the *T. gondii* B1 gene (forward primer 5′-AAC GGG CGA GTA GCA CCT GAG GAG-3′ and reverse primer 5′-TGG GTC TAC GTC GAT GGC ATG ACA AC-3′) by qPCR, as previously described (Li et al., 2020). A standard curve was constructed using 10-fold serial dilutions of *T. gondii* DNA extracted from 10^5^ parasites; thus, the curve ranged from 0.01 to 10,000 parasites. The parasite number was calculated from the standard curve.

### Statistical Analysis

To graph and analyze the data, GraphPad Prism 7 software (GraphPad Software Inc., United States) was used. In this study, statistical analyses were performed using unpaired Student’s *t*-test, Tukey’s Multiple Comparison Test, and one-way ANOVA plus Tukey-Kramer *post hoc* analysis. Data represent the mean ± Standard Error of Mean. Survival curves were generated using the Kaplan–Meier method and statistical comparisons were made by the log-rank method. A *P* value < 0.05 was considered statistically significant.

## Results

### Δ*gra9* Generation in Type II PLK Strain Markedly Reduces Cyst Formation *in vitro* and *in vivo*

**FIGURE 1:**
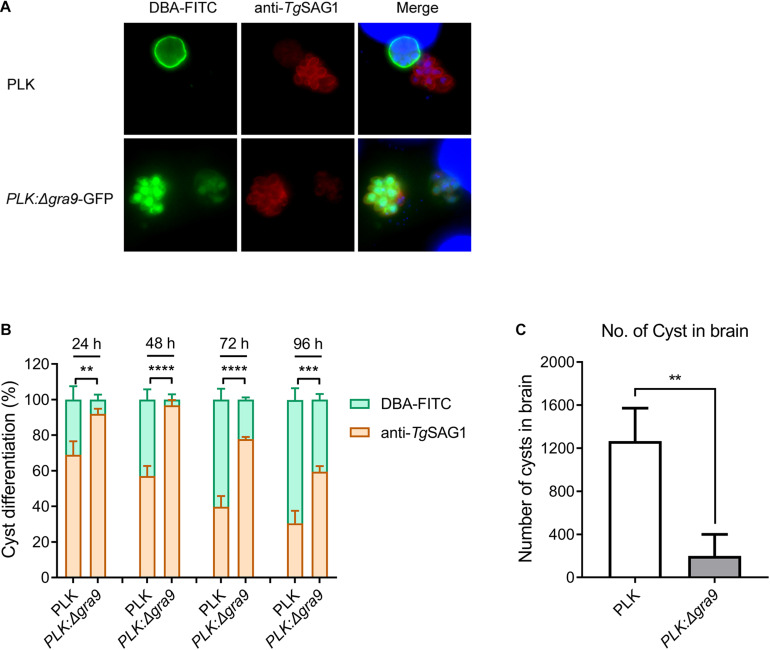
Disruption of gra9 in type II PLK strain reduces cyst formation *in vitro* and *in vivo*. **(A)** The bradyzoite differentiation from wild-type or Δ*gra9* with GFP tachyzoites under bradyzoite-inducing conditions *in vitro*. DBA-FITC (green) was used for cyst wall staining and anti-SAG1 (red) antibody was used for tachyzoite marker. Both strains formed cysts, as cysts were stained by DBA-FITC. The blue fluorescence showed the nuclear DNA staining by DAPI. **(B)** Conversion rates *in vitro*. Bradyzoite differentiation rates were counted in at least 100 vacuoles at 24, 48, 72, and 96 post-induced hours. The data are presented as the mean ± SEM of at least three independent experiments (**P < 0.01; ***P < 0.001; ****P < 0.0001; one-way ANOVA plus Dunnett’s multiple comparisons test). **(C)** The cyst number in the mouse brains of Δ*gra9* infection. The dose of 10^3^ WT or Δ*gra9* tachyzoites were injected each mouse and were monitored for 35 days, and then the number of cysts in survival mouse brains were determined (*n* = 4; **P < 0.01; Student’s *t*-test).

Our previous study revealed that (Guo et al., 2019). To see whether a single mutant of Δ*gra9* affects bradyzoite differentiation from tachyzoite, WT or Δ*gra9* (with GFP) tachyzoites were cultured under bradyzoite-inducing conditions to count the conversion rates. Both strains formed cysts, as cysts were stained by DBA-FITC (green) (Figure 1A). However, compared with the cyst differentiation rate of WT parasites, Δ*gra9* reduced by 74.19, 94.04, 63.07, and 41.52% at 24, 48, 72, and 96 h post-induction, respectively (Figure 1B). *In vivo*, with the dose of 10^3^ tachyzoites infections, no mortalities were recorded until day 35 in mice infected with parasites either strains (data not shown), while as shown in Figure 1C, the cyst number in the brain of mice with Δ*gra9* infection significantly reduced by 73.68% compared with that of the PLK. Collectively, the loss of gra9 in type II PLK strain, did not abolish, but markedly reduced cyst formation *in vitro* and *in vivo*.

### Δ*gra9* Generation in Type II PLK Strain Severely Attenuates Virulence in Mice

**FIGURE 2:**
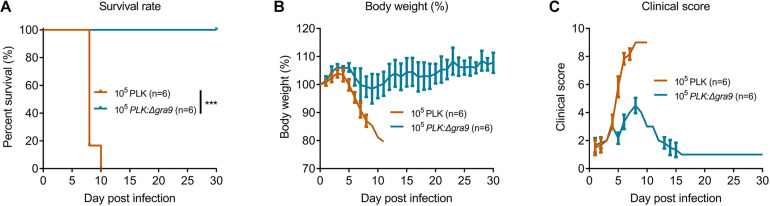
Δ*gra9* generation in PLK strain severely attenuates the virulence in mice. A lethal dose of 10^5^ parasites of wild-type or Δ*gra9* were infected into female BALB/c mice by intraperitoneal injection (*n* = 6), and mice were monitored for 30 days to note daily observations of body weight and clinical signs. **(A)** Survival rates (***P < 0.001; Log-rank (Mantel-Cox) test). **(B)** Body weight%. **(C)** Clinical scores. The scores varied from 0 (no signs) to 10 (all signs).

To evaluate the virulence of Δ*gra9*, a lethal dose of 10^5^ parasites was infected into mice by i.p., with body weight and clinical signs monitored for 30 days. The parental PLK strain caused 100% mortality (Figure 2A) with severe body weight losses (Figure 2B) and clinical symptoms (Figure 2C), whereas infection with the mutant strain surprisingly proved to be unfatal to mice (Figure 2A), indicating that Δ*gra9* generation in type II PLK strain severely attenuated virulence in mice.

### Δ*gra9* Infection Shows Different Cytokine Productions and Relatively High *T. gondii*-Specific IgG Levels Compared With Wild-Type Infection in Mice

Lethal type II strain infections are associated with extremely elevated pro-inflammatory cytokine levels in the serum, including IFN-γ and IL-12 (Mordue et al., 2001). In order to elucidate the differences in immune response caused by infection with Δ*gra9* or WT strains, mice were infected with a non-lethal dose of 10^3^ parasites. Sera were collected at 7 or 30 dpi to measure the changes in immune response. Levels of pro-inflammatory cytokines IFN-γ and IL-12, as well as anti-inflammatory cytokine IL-10, were significantly elevated at 7 and 30 dpi in both Δ*gra9* and WT infections compared with control (Supplementary Figures S1A–C). However, Δ*gra9*-infected mice showed 39.17–54.05% lower serum cytokine levels compare with WT infections at 7 dpi, although not statistically significant (Supplementary Figures S1A–C). This suggests that loss of gra9 in PLK parasites led to milder cytokine productions resulting in complete mouse survival. Conversely, similarly high *T. gondii*-specific IgG levels were induced at 30 dpi after either PLK or mutant parasite infection (Supplementary Figure S1D). At the cellular level, cytokine productions by splenocytes after *T. gondii* antigen stimulation was determined at 35 dpi, found that high levels of cytokine IFN-γ and IL-10 in Δ*gra9*-infected splenocytes were induced quickly, which were slightly higher than the WT (Supplementary Figures S1E,F). Together, these results suggest that Δ*gra9* infection induces balanced cytokine productions and relatively high *T. gondii*-specific IgG levels compared with WT infection in mice, which relates to the severely attenuated virulence of Δ*gra9* strain.

### Δ*gra9* Vaccination Induces Full *T. gondii*-Specific Immune Response

**FIGURE 3:**
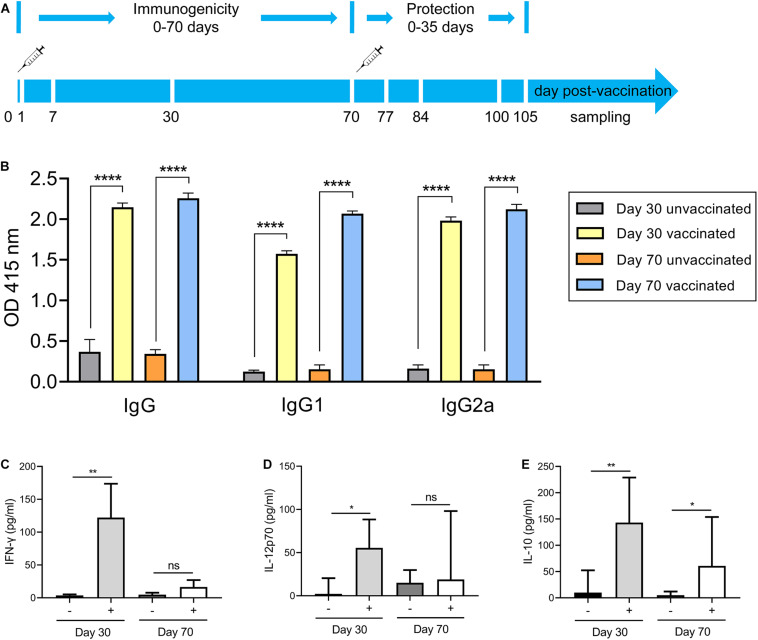
Immunization scheme and study design. **(A)** Immunization scheme. The immune dose of 10^3^ Δ*gra9* tachyzoites each mouse was injected into female BALB/c mice (*n* = 6 each group) by intraperitoneal injection and that day was designated as 0 day post-vaccination. The immunogenicity of Δ*gra9* vaccination was tested via the detection of specific anti-*T. gondii* IgG levels and cytokine productions in sera at 30 and 70 dpv. At 70 dpv, vaccinated or unvaccinated mice were secondly challenged by acute or chronic *T. gondii* infection to assess the protection. Within the vaccination and challenge period of total 105 days, the daily body weights, clinical signs, and survival rates of all mice were recorded in detail, and peritoneal fluid or serum samples were collected to evaluate immune response at the limited sampling periods as shown in **(A)**. **(B)** The specific anti-*T. gondii* IgG and IgG subclasses (IgG1 and IgG2a) levels in vaccinated mice at 30 or 70 dpv. Unvaccinated naïve mice with the same ages were used as control (****P < 0.0001; Student’s *t*-test). **(C–E)** Cytokine productions in sera at 30 and 70 dpv. The levels of IFN-γ **(C)**, IL-12p70 **(D)**, or IL-10 **(E)** were determined by ELISA kits. –, unvaccinated mice; +, vaccinated mice (*P < 0.05; **P < 0.01; ns, not significant; Student’s *t*-test).

The above findings reveal the fact that Δ*gra9* strain attenuated acute virulence and affected the formation of cysts. To evaluate the potential as a good vaccine of this mutant strain and test the immunogenicity derived from Δ*gra9* vaccination, we designed an immunization scheme within 105 days using mouse models (Figure 3A). At the outset, the chosen immune dose was 10^3^ Δ*gra9* tachyzoites each mouse by i.p. Results of our preliminary experiments showed that the lowly clinical score was observed in 10^3^ Δ*gra9*-infected mice, whereas 10^4^ and 10^5^ Δ*gra9*-infected or 10^3^, 10^4^, and 10^5^ PLK-infected mice showed severe clinical signs (Supplementary Figure S2). At 30 dpi, induced *T. gondii*-specific IgG in sera of 10^3^, 10^4^, and 10^5^ Δ*gra9*- or 10^3^ and 10^4^ PLK-infected mice were of similarly high levels (Supplementary Figure S3). These represent similar immunogenicity and suggest that Δ*gra9* vaccination of 10^3^ tachyzoites was a safe and effective immune dose. After vaccination of 10^3^ Δ*gra9* tachyzoites, the immunogenicity of Δ*gra9* was tested through detection of specific anti-*T. gondii* IgG and IgG subclasses (IgG1 and IgG2a) levels in vaccinated mouse sera at 30 and 70 dpv. Unvaccinated (naïve) mice were used as control. The ELISA results showed that Δ*gra9* vaccination induced a significantly higher *T. gondii*-specific IgG level at 30 dpv, and maintained a similarly high level at 70 dpv (Figure 3B). Nest study was to test the levels of IgG subclasses, the results showed that compared to unvaccinated mice, the level of IgG2a in vaccinated mice was significantly higher at 30 and 70 dpv (Figure 3B). To test IgG1 level in mice, although the level was lower at 30 dpv compared with 70 dpv, it was also increased to significantly higher in the vaccinated mice both 30 and 70 dpv than unvaccinated mice (Figure 3B). These suggest that Δ*gra9* vaccination elicits a mixed Th1/Th2 immune response both 30 and 70 dpv. Opposite to the stable IgG levels, cytokine levels changed over time. Relatively higher levels of pro-inflammatory IFN-γ and IL-12, or anti-inflammatory IL-10 were recorded from vaccinated mice compared with unvaccinated mice at 30 dpv, while levels decreased at 70 dpv (Figures 3C–E). Collectively, these results reveal that Δ*gra9* vaccination provided a benign humoral and cellular immune response in mice and proved to induce effective immunogenicity.

**FIGURE 4:**
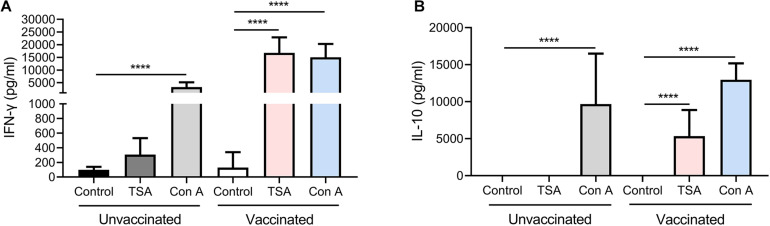
Δ*gra9* vaccination activates the ability of the splenocytes to rapidly and specifically recognize the *Toxoplasma* antigen to induce high-level cytokines, compared with unvaccinated. Immunological memory of mice in Δ*gra9* vaccination was evaluated at 70 dpv via stimulated splenocytes by total *Toxoplasma* soluble antigen resulting in the production of cytokines IFN-γ **(A)** or IL-10 **(B)**. RPMI 1640 with 20% FBS only or 5 μg/ml concanavalin A were used as negative or positive controls, respectively. The data are presented as the mean ± SEM of at least three repeats each sample (****P < 0.0001; Student’s *t*-test).

To assess the immunological memory in Δ*gra9* vaccinated mice, splenocytes were harvested from vaccinated or unvaccinated mice at 70 dpv, and stimulated with total *Toxoplasma* soluble antigen (TSA) prepared from fresh wild-type (PLK) tachyzoites. As shown in Figures 4A,B, the significantly high levels of pro-inflammatory cytokine IFN-γ, as well as anti-inflammatory cytokine IL-10, were stimulated by TSA compared with no stimulation or no vaccination. Interestingly, although the significantly decreased levels of stimulated IFN-γ (16,787.5 pg/ml) and IL-10 (5,347.5 pg/ml) at 70 dpv were observed compared with their levels of IFN-γ (207,667.7 pg/ml) and IL-10 (10,210.4 pg/ml) at 30 dpv in the Δ*gra9* vaccinated splenocytes (Supplementary Figures S1E,F), there were obviously high levels at both time points. These suggest that Δ*gra9* vaccination could activate the ability of the immune cells to quickly and specifically recognize the *Toxoplasma* antigen to produce corresponding immune cytokines in short-term and long-term immunization.

### Δ*gra9* Vaccination Confers Potent for Protection Against Acute and Chronic *T. gondii* Challenges

**FIGURE 5:**
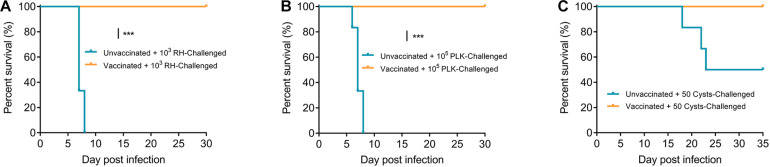
Δ*gra9* vaccination protects mice against *T. gondii* infections. Vaccinated or unvaccinated mice were challenged with 10^3^ type I RH **(A)** or 10^5^ type II PLK **(B)** tachyzoites by intraperitoneal injection or 50 PLK cysts **(C)** by oral administration at 70 dpv, and monitored for another 30 days for tachyzoite or 35 days for cyst infections to note survival rates (***P < 0.001; Log-rank (Mantel-Cox) test).

Based on the findings that Δ*gra9* has strong immunogenicity and immunological memory, we then preformed the second challenges with 10^3^ type I (RH) or 10^5^ type II (PLK) tachyzoites by i.p. or 50 cysts (PLK) by oral administration to vaccinated mice at 70 dpv. All challenged mice were monitored for another 30 days for tachyzoite or 35 days for cyst infections, and recorded daily body weights, clinical signs, and survival rates. For both RH (Figure 5A) and PLK (Figure 5B) tachyzoite challenges, Δ*gra9* vaccination elicited strong protection following 100% survival rates of mice and no obvious signs or body weight changes were observed during the 30-day challenge period (Supplementary Figure S4). However, for unvaccinated mice challenged with the lethal dose of RH or PLK tachyzoites, the clinical signs and body weight changes were initially observed at day 4 of RH or day 2 of PLK infections until the signs developed to most severe at 6-8 days (Supplementary Figure S4), resulting in 100% mortality rates for unvaccinated mice within 8 days post-challenges (Figures 5A,B). As expected, all Δ*gra9*-vaccinated mice survived when infected with 50 cysts (PLK strain), whereas only 50% of unvaccinated mice survived (Figure 5C). While the 50 cysts-challenges to vaccinated or unvaccinated mice led to decreased body weights (%) in both groups during the whole period of 35 days, clinical signs were observed only in unvaccinated mice starting from day 8 post-challenge infection (Supplementary Figure S4). Altogether, Δ*gra9* vaccination confers strong protective immunity against acute and chronic toxoplasmosis.

### Δ*gra9* Vaccination Rapidly Clears Challenging Parasites and Blocks Cyst Formation in New Challenges

**FIGURE 6:**
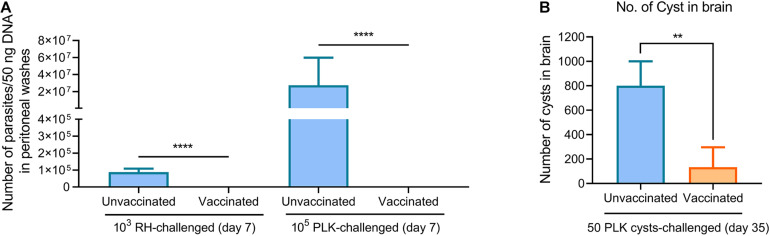
Δ*gra9* vaccination rapidly clears challenging parasites and blocks cyst formation in new challenges. **(A)** Parasite burdens in peritoneal fluids of RH or PLK tachyzoites-challenged vaccinated mice. A quantitative PCR of the *Tg*B1 gene using 50 ng extracted DNA from peritoneal fluids was used to confirm parasite proliferation in vaccinated mice at 7 days post-challenge infection, compared with unvaccinated challenged mice (****P < 0.0001; Student’s *t*-test). **(B)** No. of cyst in brain in PLK cysts-challenged vaccinated mice. At day 35 post-challenges, the brains were isolated from survival mice of 50 cysts challenges to estimate cyst numbers by DBA-FITC staining (**P < 0.01; Student’s *t*-test).

To further understand how Δ*gra9* vaccination provided strong protection in mice suffering both acute and chronic *T. gondii* challenges, peritoneal fluids and sera in challenged mice were collected at 7 days tachyzoite post-challenges or 14 days cyst post-challenges (time-points when we observed the most serious signs in unvaccinated mice) to determine cytokine productions, as well as parasite burdens in peritoneal fluids. In naïve mice, RH infections resulted in rapid proliferation with 87,305 parasites in 50 ng DNA, whereas PLK infections caused higher parasite burdens of 2.74 × 10^7^ at 7 days post-challenge infection (Figure 6A). However, we could not detect any parasites using a qPCR test in both RH- and PLK- challenged Δ*gra9*-vaccinated mice, suggesting that Δ*gra9* vaccination promoted the activity to rapidly clear infecting parasites. Meanwhile, for chronic toxoplasmosis, parasites were not detected in any mice peritoneal fluid at 14 dpi (data not shown), but markedly reduced number of cysts in vaccinated survival mouse brain was noted by day 35 compared with unvaccinated mice (Figure 6B), which are similar to that level of cyst formation of vaccinated but no cyst challenged mice (Figure 1C). This suggests that Δ*gra9* vaccination blocks cyst formation in new challenged cysts.

**FIGURE 7:**
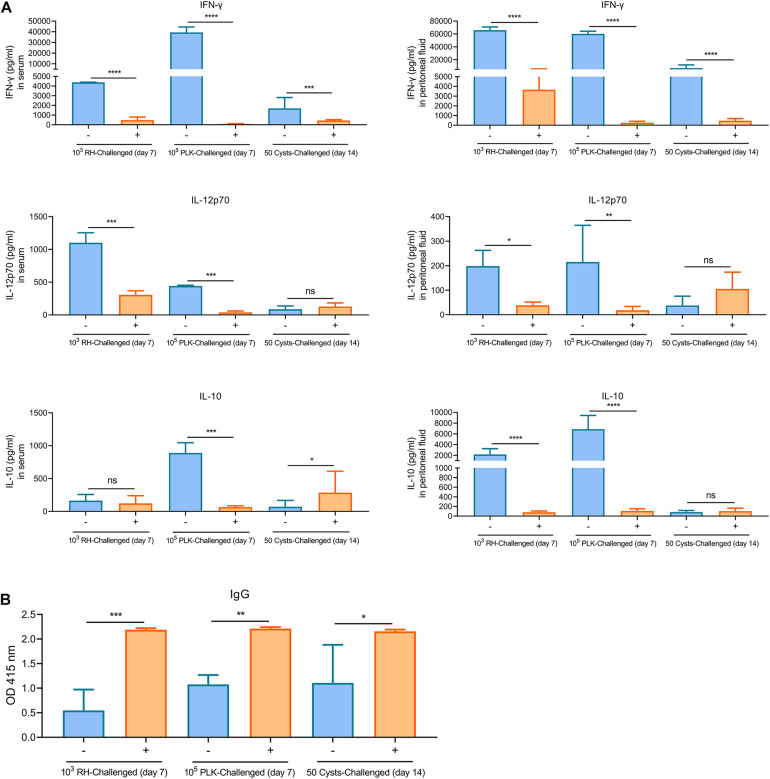
Δ*gra9* vaccination provides safe and effective immune protection. The peritoneal fluids and sera in challenged mice were collected at 7 days tachyzoite post-challenges or 14 days cyst post-challenges to determine cytokine productions and *T. gondii* specific IgG, compared with unvaccinated but secondly challenged mouse samples. **(A)** IFN-γ, IL-12p70, or IL-10 levels in serum or peritoneal fluid samples. –, unvaccinated; +, vaccinated (*P < 0.05; **P < 0.01; ***P < 0.001; ****P < 0.0001; ns, not significant; Student’s *t*-test). **(B)** The levels of *T. gondii* specific IgG. IgG levels in vaccinated challenged or unvaccinated challenged mice at 7 days tachyzoite post-challenges or 14 days cyst post-challenges were determined by an ELISA test. –, unvaccinated, +, vaccinated (*P < 0.05; **P < 0.01; ***P < 0.001; Student’s *t*-test).

Subsequently, the immune responses were also tested during the limited time-points, levels of cytokines (IFN-γ, IL-12, and IL-10) in both peritoneal fluid and serum were substantially induced in unvaccinated mice relative to the extremely low levels in Δ*gra9*-vaccinated mice, especially the IFN-γ levels (Figure 7A). More importantly, all mice remained with high levels of *T. gondii*-specific IgG (Figure 7B). Taken together, Δ*gra9* vaccination provided efficient and safe immune protection to kill challenging parasites, resulting in host survival.

### A Special Protection Against *T. gondii* Infection Is Provided by the Sera of Δ*gra9*-Vaccinated Mice

**FIGURE 8:**
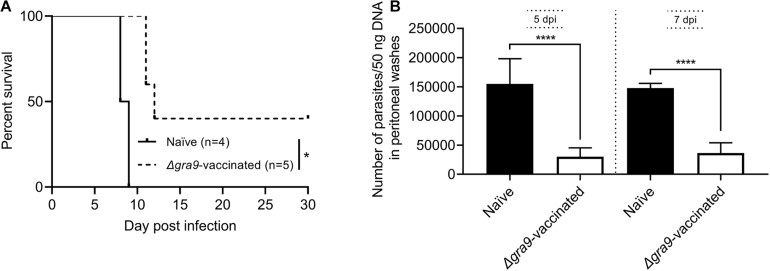
The sera from Δ*gra9*-vaccinated mice are able to reduce parasite propagation in mice. The sera from *PLK*:Δ*gra9*-vaccinated mice were administered into 10^5^ PLK parasites infected mice at 0 and 3 day post-infection, survival rates were noted and parasite burdens were determined in peritoneal fluids at day 5 and 7 post-infection to evaluate parasite growth under passive immunization. The four mice for naïve sera as negative control, and five mice for *PLK*:Δ*gra9*-vaccinated sera as test group. **(A)** Survival rates (*P < 0.05; Log-rank (Mantel-Cox) test). **(B)** Parasite burdens (****P < 0.0001; Student’s *t*-test).

Δ*gra9* vaccination induced a significantly high *T. gondii*-specific IgG level with low cytokine levels at 70 dpv in mouse sera as shown above. In this study, the sera from *PLK*:Δ*gra9*-vaccinated mice were administered into parasites infected mice with lethal dose, survival rates were recorded and parasite burdens were determined in peritoneal fluids at day 5 and 7 post-infection to evaluate parasite growth under passive immunization. The results showed that vaccinated sera gave 40% survival rates (*n* = 5, Figure 8A) and led to significantly lower parasite burdens in peritoneal fluids of WT parasites-infected mice both 5 and 7 dpi, compared with naïve sera (Figure 8B). As shown in Figure 8B, the numbers of parasite in control mice at 5 or 7 day post-infection were resulted in 5 or 4 times of mice injected with vaccinated sera, respectively. These suggest that the sera of Δ*gra9*-vaccinated mice with high IgG and low cytokine levels are able to reduce parasite propagation in mice.

## Discussion

In recent years, active immunization is considered to be the ideal and long-term strategy to induce the host immune response against acute and chronic *T. gondii* infections (Loh et al., 2019). One means is to develop live attenuated vaccines, a whole parasite-based vaccine, which is the live strain with reduced replication and attenuated virulence but retaining the ability to induce an immune response against a variety of wild-type strain and multiple stages of parasite infections (Wang et al., 2017, 2018; Xia et al., 2018b; Yang et al., 2019; Liang et al., 2020). In this study, *PLK*:Δ*gra9* vaccination induced a high level of immune response and resisted the infections of tachyzoites from type I or type II strains and cysts from type II strain.

Our previous study reported that the generation of mutant Δ*gra9* strain in PLK parasites not only significantly reduced the growth of tachyzoites *in vitro* but also changed replication in mice resulting in severely attenuated virulence (Guo et al., 2019), which consistent with the current result of 100% survival rate in mice during challenge infection with 10^5^ Δ*gra9* parasites which have been passed for more than 250 generations in culture cells. This suggests that the attenuated *Toxoplasma* strain did not revert back to virulent form in animals at least 250 cell passages under confirmed loss of gra9 gene. Importantly, GRAs have been investigated with playing the major structural roles within the PV and the cyst wall of *T. gondii* (Guevara et al., 2019). Current results demonstrate that loss of gra9 in type II strain (PLK), reduced the formation of cysts *in vitro* and *in vivo*, which corroborates gra9 protein as one of the intravacuolar-network-associated dense GRAs involved during cyst development (Fox et al., 2019; Guevara et al., 2019). To test the virulence of cysts, we isolated and injected 50 cysts of Δ*gra9* were into three mice which resulted in no mice deaths (data not shown), suggesting the possibility that the loss of gra9 in type II PLK strain might reduce the virulence of cysts, although there was limited little number of mouse model. Δ*gra9* strain’s inability to form cysts or significantly reduce cyst formation and the lack of virulence from the type II parasites are good indicators that the produced strain could be utilized as a candidate vaccine. On the other hand, compared with the severe immune response after wild-type parasite infection, Δ*gra9*-infected mice produced moderate immune response which represents a balance between inducing pro-inflammatory and anti-inflammatory cytokines, denoting its safety as a live attenuated vaccine. Furthermore, it is worthy of attention that the chosen immune dose was 10^3^ Δ*gra9* tachyzoites each mouse, since mice with 10^3^ Δ*gra9* infection observed the high specific anti-*T. gondii* IgG levels and no significantly clinical symptoms compared with naïve mice, suggesting that the vaccination with 10^3^ Δ*gra9* tachyzoites was effective and safe immune dose.

In the two phases of the entire experiment, the generation of immunogenicity and the second challenge of protection, we recorded in detail the changes in the levels of cytokines and antibodies in mice. Compared with unimmunized mice, the vaccinated mice showed significantly higher levels of the pro-inflammatory cytokines IFN-γ and IL-12 and the anti-inflammatory cytokine IL-10 at 30 dpv and dropped to low levels at 70 dpv. In the subsequent challenge infection, cytokine levels of vaccinated mice at 7 days post-challenge infection surprisingly remained at similar levels as before being challenged, suggesting the second challenges did not cause severe immune responses in vaccinated mice. Importantly, the specific anti-*T. gondii* IgG was maintained at a similarly high level throughout the process. While to test the levels of IgG subclasses, the results confirmed that both IgG1 and IgG2a in vaccinated mice was significantly higher levels at challenged time-point compared with unvaccinated mice. These suggest that Δ*gra9* vaccination elicits a mixed Th1/Th2 immune response at this time-point. On the other hand, the passive immunization test was observed that the sera from vaccinated mice could reduce parasite propagation. Hence, we consider that the Δ*gra9* vaccination-induced full protective immunity was a cellular immunity-based immune response combined with humoral immunity. At 70 dpv, Δ*gra9* vaccination induced a Th1-biased inflammatory response to produce IFN-γ and IL-12 which are two pro-inflammatory cytokines crucial for activation of cell-mediated immunity against *T. gondii* infection (Pifer and Yarovinsky, 2011; Hunter and Sibley, 2012; Yarovinsky, 2014). Meanwhile, the anti-inflammatory cytokine IL-10 was also highly produced suggesting that the balanced level of cytokines was maintained in Δ*gra9*-vaccinated mice. This balance was also proved by the splenocytes stimulation test, which rapidly and specifically produced significantly high levels of pro-inflammatory cytokine IFN-γ and anti-inflammatory cytokine IL-10. When vaccinated mice are secondly challenged with wild-type parasites, the immune system rapidly recognizes and produces high levels of IFN-γ and other pro-inflammatory cytokines to activate cellular immune responses, and simultaneously, the high level of *T. gondii-*specific IgG antibodies hinders the parasite attachment and invasion to host cells and activates the classical complement pathway to clear secondary infections efficiently in cooperation (Sayles et al., 2000; Spellberg and Edwards, 2001; Pifer and Yarovinsky, 2011; Hunter and Sibley, 2012). These are consistent with our results that Δ*gra9* vaccination rapidly blocks challenging parasite tachyzoites and new cyst formation.

In the present mouse models, noteworthy is the lack of any obvious signs or body weight changes in vaccinated mice during challenge periods, signifying that infecting parasites are strongly inhibited from replication and killed quickly by the rapid immune response during tachyzoite challenges. This might also explain why the increased levels of cytokine productions were not observed at day 7 post-challenge. In contrast, we noted significantly high parasite burden and the high levels of pro-inflammatory or anti-inflammatory cytokines with severe clinical symptoms and significant weight loss resulting in 100% mortality in naïve mice challenge infected with a lethal dose of tachyzoites. On the other hand, the Δ*gra9*-vaccination-induced full immune response was not only observed against type I RH and type II PLK wild-type tachyzoite acute infections but also against type II cyst challenge and reduction of cyst formation. Interestingly, IFN-γ was cytokine which had significantly different levels between vaccinated and unvaccinated mice at day 14 post-cyst-challenge, suggesting that IFN-γ is also central to the development of immunity against *T. gondii* cyst infection, consistent with Δ*gra9*-vaccination-induced immune response for protection of cyst challenge.

## Conclusion

In conclusion, the present study demonstrated that loss of gra9 in type II PLK strain dramatically attenuated virulence and significantly reduced the formation of cysts in animals. Δ*gra9* vaccination effectively elicited immune responses which conferred absolute protection from subsequent lethal type I RH, type II PLK wild-type tachyzoites or type II PLK cysts challenge infections in mice, suggesting that the mutant Δ*gra9* of *T. gondii* type II PLK strain is a potential live-attenuated vaccine candidate against acute and chronic toxoplasmosis. However, the current study is limited to the mouse model; thus, the effectiveness and safety should likewise be extensively investigated in animals of veterinary and economic importance, including but not limited to definitive hosts cats, susceptible sheep and pigs and other meat-producing animals. Future studies should assess its effectiveness against other *Toxoplasma* strains, particularly infections with local endemic strains.

## Data Availability Statement

The raw data supporting the conclusions of this article will be made available by the authors, without undue reservation.

## Ethics Statement

The animal study was reviewed and approved by the Committee on the Ethics of Animal Experiments at the Obihiro University of Agriculture and Veterinary Medicine, Japan (permission numbers: 190246).

## Author Contributions

JL and XX conceived and designed the experiments. JL, EG, HG, ML, YL, and SJ performed the experiments. JL, IZ, YG, and WZ analyzed the data and wrote the manuscript. PA, MR, MT, BB, AR, and TM critically revised the manuscript. All authors read and approved the final version of the manuscript.

## Conflict of Interest

The authors declare that the research was conducted in the absence of any commercial or financial relationships that could be construed as a potential conflict of interest.

## Supplementary Material

Supplementary Figure 1 Δ*gra9* infection induces mild cytokine productions and relatively high *T. gondii*-specific IgG levels in mice compared with Wild-type PLK infection. A non-lethal dose of 10^3^ parasites was chosen to infect BALB/c mice, and the sera were collected at day 7 or 30 post-infection to measure immune response. Naïve mouse sera were used as controls. The levels of pro-inflammatory cytokines IFN-γ **(A)** and IL-12p70 **(B)** or anti-inflammatory cytokine IL-10 **(C)** were assessed by an ELISA test (*n* = 4; ^∗^*P* < 0.05; ^∗∗^*P* < 0.01; ^∗∗∗^*P* < 0.001; ^****^*P* < 0.0001, Control vs parental or Δ*gra9* at 7 dpi; ^#^*P* < 0.05, control vs parental or Δ*gra9* at 30 dpi; one-way ANOVA plus Tukey-Kramer *post hoc* analysis). **(D)** The *T. gondii*-specific IgG levels were measured at day 7 or 30 post-infection compared with naïve mice (^****^*P* < 0.0001; Student’s *t*-test). **(E,F)** Cytokine productions by splenocytes after *T. gondii* antigen stimulation. At 35 dpi, the splenocytes were harvested from parental or Δ*gra9* infected mouse spleens, and 3 × 10^5^ viable splenocytes were cultured *in vitro* and stimulated by *T. gondii* antigen to induce cytokine IFN-γ **(E)** or IL-10 **(F)** productions. For the negative and positive controls, the same number of splenocytes was cultured at same time and stimulated with RPMI 1640 with 20% FBS only or 5 μg/ml concanavalin A, respectively (^****^*P* < 0.0001; Student’s *t*-test).

Supplementary Figure 2 Clinical symptoms of mice during parasite infection. The different dose of 10^3^, 10^4^, and 10^5^Δ *gra9*- or 10^3^, 10^4^, and 10^5^ PLK-parasites were injected into mice, and clinical signs of infected mice were noted within 30 days post-infection. The clinical scores varied from 0 (no signs) to 10 (all signs). No-infected mice were used as control.

Supplementary Figure 3 *T. gondii*-specific IgG in sera from *Toxoplasma* infected-mice. The different dose of 10^3^, 10^4^, and 10^5^ Δ*gra9*- or 10^3^ and 10^4^ PLK-parasites were injected into mice, at 30 dpi, induced *T. gondii*-specific IgG in sera were detected by ELISA tests. No-infected sera and PBS only were used as control (^****^*P* < 0.0001; Student’s *t*-test).

Supplementary Figure 4 Δ*gra9* vaccination protects mice against *T. gondii* infections. Vaccinated or unvaccinated mice were challenged with 10^3^ type I RH or 10^5^ type II tachyzoites by intraperitoneal injection or 50 PLK cysts by oral administration at 70 dpv, and monitored for another 30 days for tachyzoite or 35 days for cyst infections to note daily body weights **(A–C)** and clinical signs **(D–F)** in detail.
