# Supplementary figures and images for: PLK:Δgra9 Live Attenuated Strain Induces Protective Immunity Against Acute and Chronic Toxoplasmosis
Source: Front Microbiol. 2021 Mar 11;12:619335. doi: 10.3389/fmicb.2021.619335 (PMC7991750; doi:10.3389/fmicb.2021.619335)

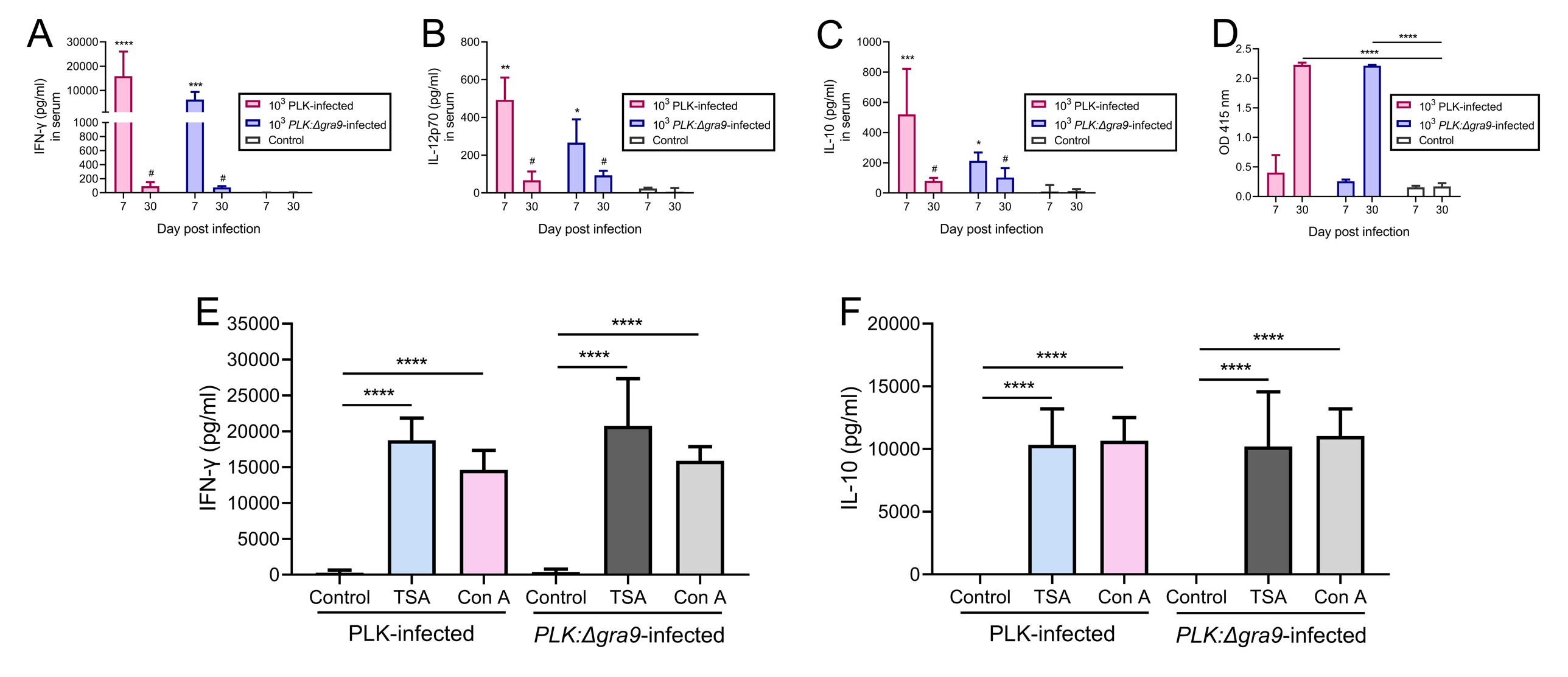

Supplement: Supplementary Figure 1 — Δgra9 infection induces mild cytokine productions and relatively high T. gondii-specific IgG levels in mice compared with Wild-type PLK infection. A non-lethal dose of 103 parasites was chosen to infect BALB/c mice, and the sera were collected at day 7 or 30 post-infection to measure immune response. Naïve mouse sera were used as controls. The levels of pro-inflammatory cytokines IFN-γ (A) and IL-12p70 (B) or anti-inflammatory cytokine IL-10 (C) were assessed by an ELISA test (n = 4; ∗P < 0.05; ∗∗P < 0.01; ∗∗∗P < 0.001; ****P < 0.0001, Control vs parental or Δgra9 at 7 dpi; #P < 0.05, control vs parental or Δgra9 at 30 dpi; one-way ANOVA plus Tukey-Kramer post hoc analysis). (D) The T. gondii-specific IgG levels were measured at day 7 or 30 post-infection compared with naïve mice (****P < 0.0001; Student’s t-test). (E,F) Cytokine productions by splenocytes after T. gondii antigen stimulation. At 35 dpi, the splenocytes were harvested from parental or Δgra9 infected mouse spleens, and 3 × 10^5^ viable splenocytes were cultured in vitro and stimulated by T. gondii antigen to induce cytokine IFN-γ (E) or IL-10 (F) productions. For the negative and positive controls, the same number of splenocytes was cultured at same time and stimulated with RPMI 1640 with 20% FBS only or 5 μg/ml concanavalin A, respectively (****P < 0.0001; Student’s t-test). [file Image_1.TIF]

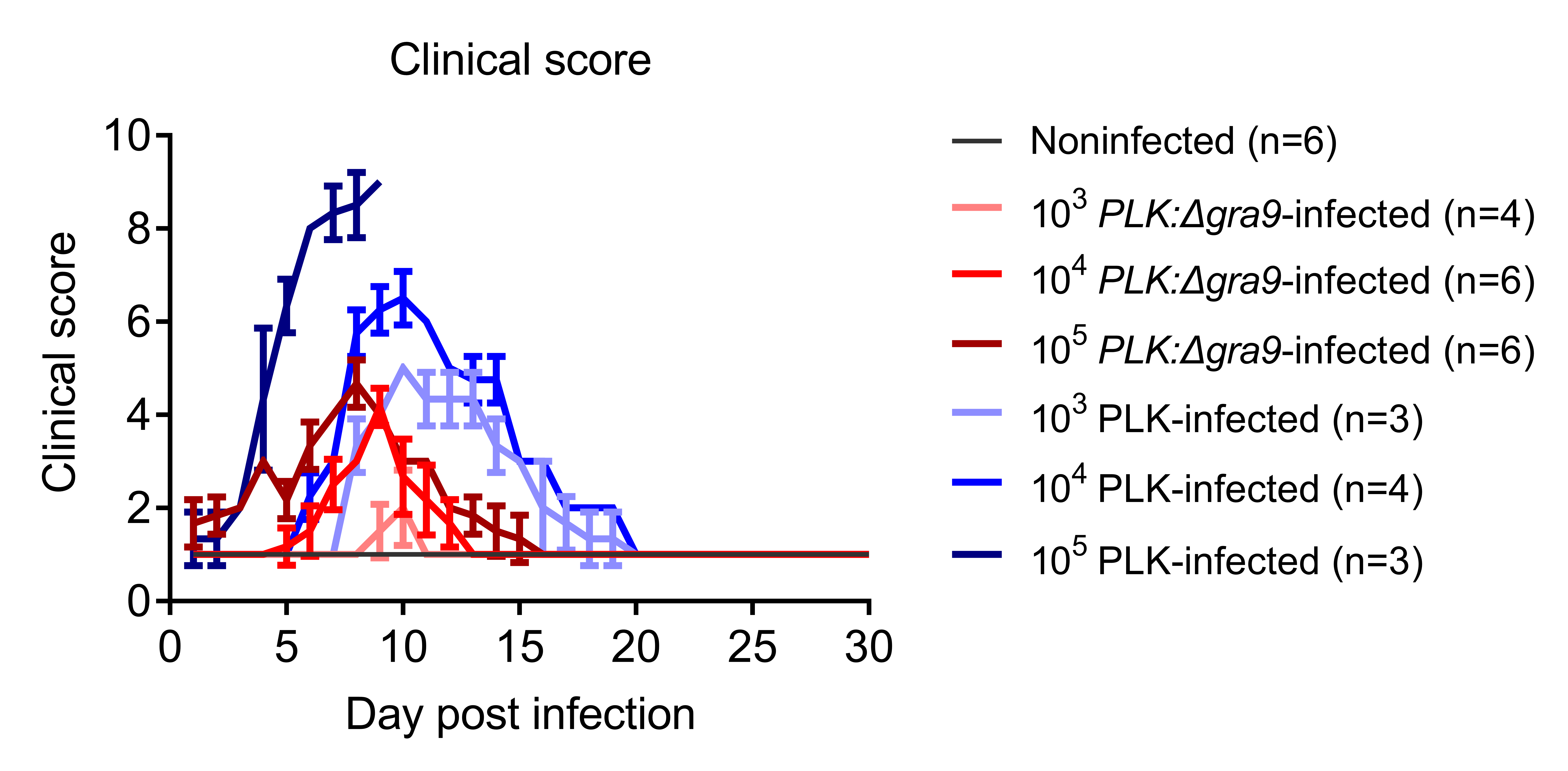

Supplement: Supplementary Figure 2 — Clinical symptoms of mice during parasite infection. The different dose of 103, 104, and 105Δ gra9- or 103, 104, and 105 PLK-parasites were injected into mice, and clinical signs of infected mice were noted within 30 days post-infection. The clinical scores varied from 0 (no signs) to 10 (all signs). No-infected mice were used as control. [file Image_2.TIF]

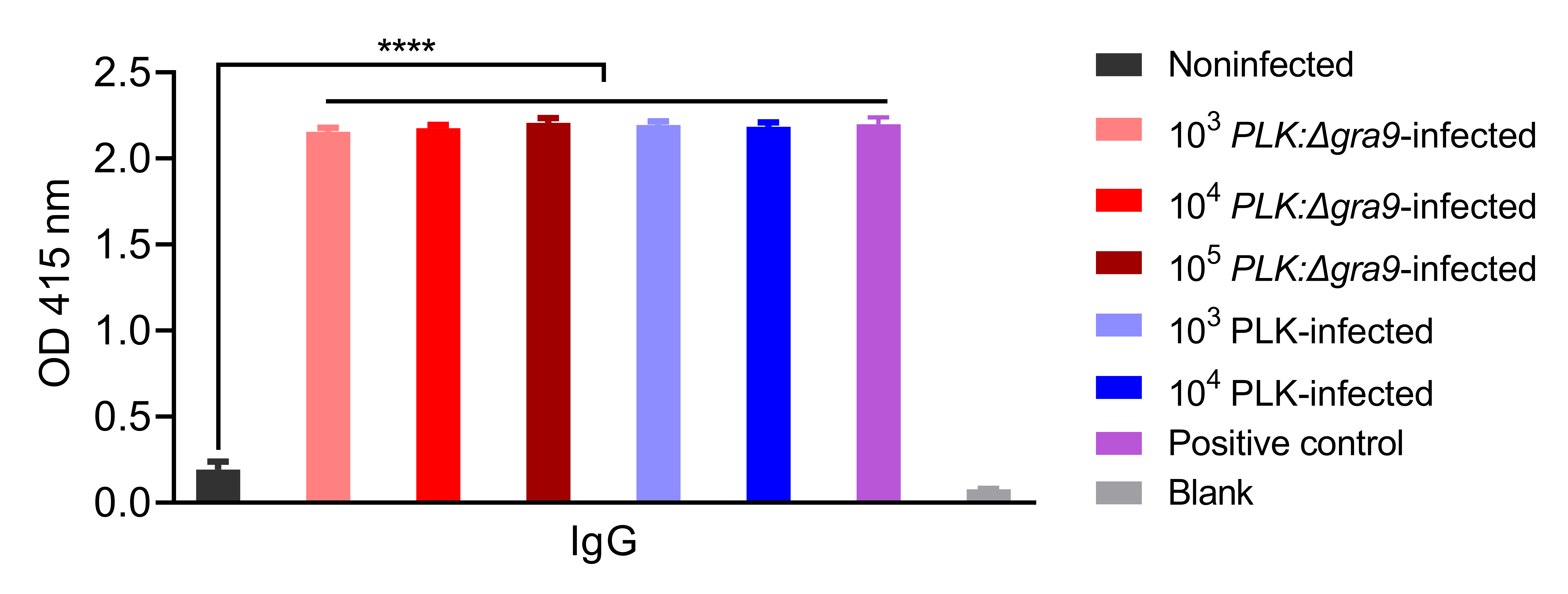

Supplement: Supplementary Figure 3 — T. gondii-specific IgG in sera from Toxoplasma infected-mice. The different dose of 103, 104, and 105 Δgra9- or 103 and 104 PLK-parasites were injected into mice, at 30 dpi, induced T. gondii-specific IgG in sera were detected by ELISA tests. No-infected sera and PBS only were used as control (****P < 0.0001; Student’s t-test). [file Image_3.TIF]

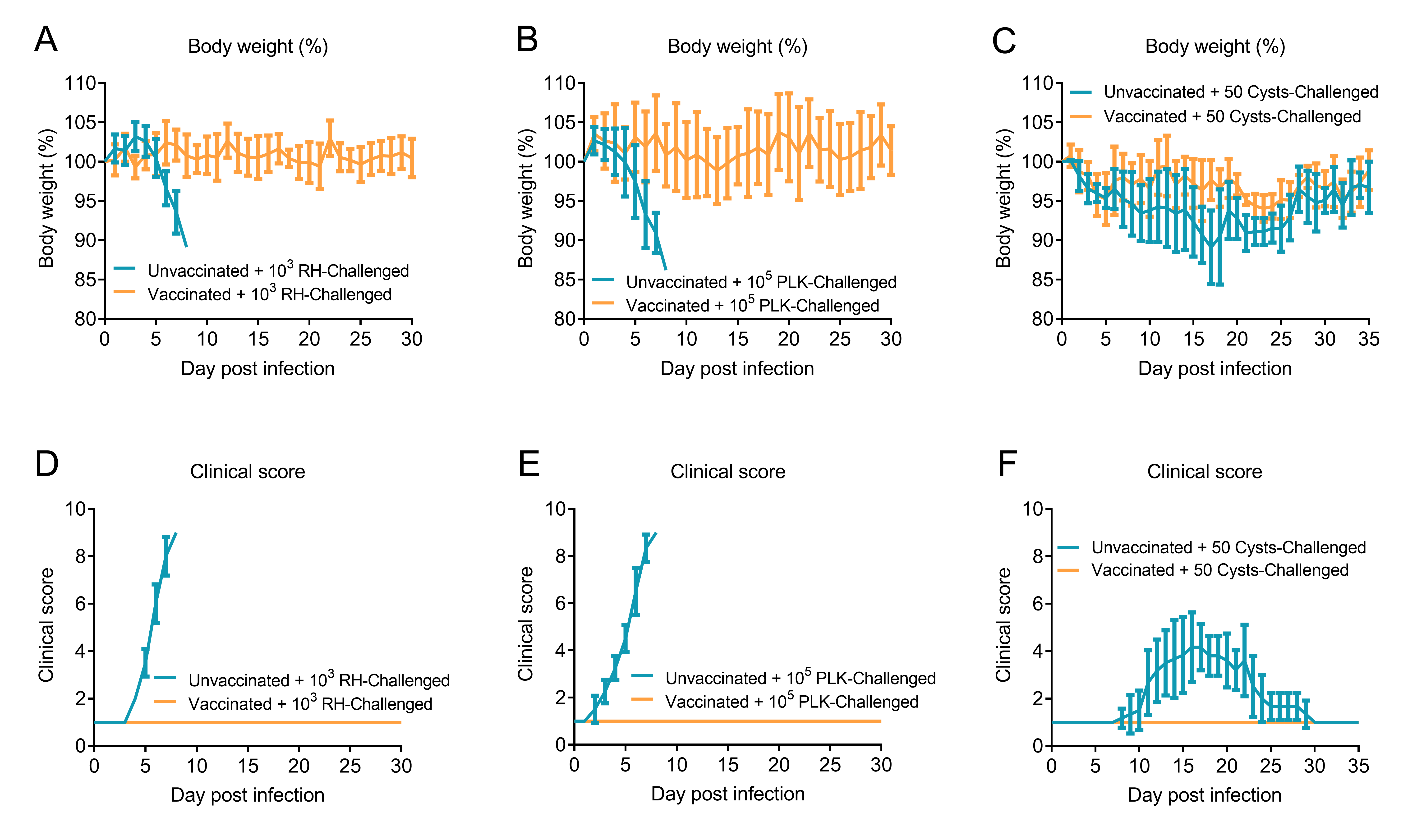

Supplement: Supplementary Figure 4 — Δgra9 vaccination protects mice against T. gondii infections. Vaccinated or unvaccinated mice were challenged with 103 type I RH or 105 type II tachyzoites by intraperitoneal injection or 50 PLK cysts by oral administration at 70 dpv, and monitored for another 30 days for tachyzoite or 35 days for cyst infections to note daily body weights (A–C) and clinical signs (D–F) in detail. [file Image_4.TIF]
